# Cetuximab Augments Cytotoxicity with Poly (ADP-Ribose) Polymerase Inhibition in Head and Neck Cancer

**DOI:** 10.1371/journal.pone.0024148

**Published:** 2011-08-30

**Authors:** Somaira Nowsheen, James A. Bonner, Albert F. LoBuglio, Hoa Trummell, Alexander C. Whitley, Michael C. Dobelbower, Eddy S. Yang

**Affiliations:** 1 Department of Radiation Oncology, Comprehensive Cancer Center, University of Alabama at Birmingham School of Medicine, Birmingham, Alabama, United States of America; 2 Department of Cell Biology, University of Alabama at Birmingham, Birmingham, Alabama, United States of America; 3 Department of Pharmacology and Toxicology, University of Alabama at Birmingham, Birmingham, Alabama, United States of America; 4 Department of Hematology/Oncology, Comprehensive Cancer Center, University of Alabama at Birmingham School of Medicine, Birmingham, Alabama, United States of America; University Medical Center Hamburg-Eppendorf, Germany

## Abstract

Overexpression of the epidermal growth factor receptor (EGFR) is a hallmark of head and neck cancers and confers increased resistance and inferior survival rates. Despite targeted agents against EGFR, such as cetuximab (C225), almost half of treated patients fail this therapy, necessitating novel therapeutic strategies. Poly (ADP-Ribose) polymerase (PARP) inhibitors (PARPi) have gained recent attention due to their unique selectivity in killing tumors with defective DNA repair. In this study, we demonstrate that C225 enhances cytotoxicity with the PARPi ABT-888 in UM-SCC1, UM-SCC6, and FaDu head and neck cancer cells. The mechanism of increased susceptibility to C225 and PARPi involves C225-mediated reduction of non-homologous end-joining (NHEJ)- and homologous recombination (HR)-mediated DNA double strand break (DSB) repair, the subsequent persistence of DNA damage, and activation of the intrinsic apoptotic pathway. By generating a DSB repair deficiency, C225 can render head and neck tumor cells susceptible to PARP inhibition. The combination of C225 and the PARPi ABT-888 can thus be an innovative treatment strategy to potentially improve outcomes in head and neck cancer patients. Furthermore, this strategy may also be feasible for other EGFR overexpressing tumors, including lung and brain cancers.

## Introduction

The epidermal growth factor receptor (EGFR) plays an essential role in carcinogenesis by modulating proliferation, differentiation, and the DNA damage response [1]–[5]. In particular, overexpression and amplification of the EGFR is present in 80–100% of squamous cell carcinomas of the head and neck and portends poor prognosis, inferior survival, radioresistance, and treatment failures [3], [6]. Thus, EGFR has become heavily targeted as a cancer therapeutic strategy, and this has improved response rates, locoregional control, and overall survival in combination with radiation in head and neck cancer patients [2], [7]. However, almost half of head and neck cancer patients treated with this strategy will still succumb to this disease. Novel strategies are thus needed to improve outcomes.

Agents which target cancers that are deficient in homologous recombination (HR)-mediated DNA double strand break (DSB) repair, such as poly (ADP-ribose) polymerase (PARP) inhibitors (PARPi), have gained recent attention due to their highly selective killing of BRCA-associated, DNA repair defective tumors while maintaining minimal toxicity in normal tissues [8]–[10]. Additionally, PARPi has been reported to enhance cytotoxicity in sporadic tumors when combined with other DNA damaging agents, such as with platinum and cyclophosphamide in breast cancer and with temozolomide in glioblastoma [11]. Thus, much effort has been undertaken to expand the utility of PARPi beyond the realm of BRCA-associated tumors by combining with agents that alter the DNA damage/repair pathways.

We and others have previously reported that targeting the EGFR pathway induces a DSB repair deficiency [4], [12]–[15]. Based on these observations, we hypothesized that cetuximab (C225), a potent inhibitor of EGFR, could increase tumor susceptibility to PARPi. In this study, and consistent with our hypothesis, we demonstrate that C225 augments cytotoxicity with the PARPi ABT-888 in UM-SCC1, UM-SCC6, and FaDu head and neck cancer cells by enhancing the intrinsic apoptotic pathway. Further dissection of the mechanism of induced cell death reveals that C225 reduces non-homologous end joining (NHEJ)- and HR-mediated DNA DSB repair, which results in the persistence of DNA damage following PARPi. By generating a DSB repair deficiency, C225 can render head and neck tumor cells susceptible to PARP inhibition. Thus, the combination of C225 and the PARPi ABT-888 can be an innovative treatment strategy to potentially improve outcomes in head and neck cancer patients. Furthermore, this strategy may also be feasible in other EGFR-dysregulated tumors, such as brain and lung.

## Results

### Cetuximab enhances cytotoxicity with PARPi

We have previously demonstrated that C225, the anti-EGFR monoclonal antibody, effectively inhibits receptor activity by blocking the ligand binding site [16]. The effect of C225 on cell viability and growth has also been well studied [17]. Studies have shown that EGFR can confer increased resistance to DNA damage by enhancing cellular DSB repair capacity. Conversely, inhibition of EGFR can inhibit DSB repair. Based on these observations, we hypothesized that C225 can enhance cytotoxicity with the PARPi ABT-888 in UM-SCC1, UM-SCC6, and FaDu cells, which are well characterized, EGFR overexpressing, representative squamous cell carcinoma of the head and neck [17]–[20].

**Figure 1:**
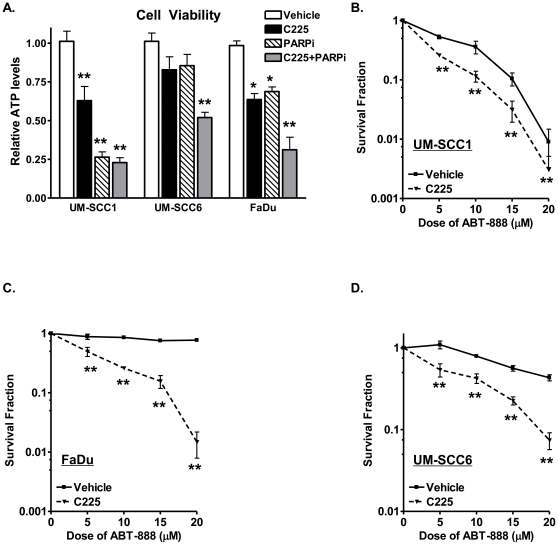
Cetuximab (C225) enhances cytotoxicity with the PARP inhibitor ABT-888 in head and neck cancer cells. (A) Combination C225 and ABT-888 reduces the viability of UM-SCC1, UM-SCC6, and FaDu head and neck cancer cells. Cells were treated with either vehicle or 2.5 µg/mL C225 for 16 hours and subsequently exposed to vehicle or 10 µM ABT-888. Twenty-four hours following ABT-888, cell viability was assayed with the ATPlite system (Perkin Elmer). Shown is the representative data of at least 3 independent experiments of the cell viability following various treatments as measured by relative ATP levels (mean +/− SEM, *p<0.01, **p<0.001 compared to vehicle control). (B–D) Combination C225 and ABT-888 reduces the colony forming ability of (B) UM-SCC1, (C) UM-SCC6, and (D) FaDu head and neck cancer cells. Cells were treated with either vehicle or 2.5 µg/mL C225 for 16 hours. Following the treatment period, cells were seeded for colony formation assays and subjected to various doses of ABT-888. Shown is the mean survival fraction (+/− SEM) from at least 3 independent colony formation assay experiments following treatment (**p<0.001).

To test this hypothesis, head and neck cancer cell viability following C225 and ABT-888 was investigated using the ATPlite assay. The doses of C225 and ABT-888 chosen have been previously reported to be within physiologic range [2], [7], [9], [21]. As shown in Fig. 1A, differential susceptibility to C225 and ABT-888 was observed in all cell lines examined (50 to 75% reduction in cell viability with combination treatment), suggesting that C225 indeed increases cell death with ABT-888. Surprisingly, UM-SCC1 cells were also susceptible to PARPi alone (approximately 75% reduction in cell viability with 10 µM ABT-888).

To confirm these findings, we also performed colony forming assays in the presence of C225 in combination with various doses (1–20 µM) of ABT-888. Consistent with the cell viability data, the addition of C225 to ABT-888 significantly reduced the colony forming ability of UM-SCC1, UM-SCC6, and FaDu cells in a dose-dependent manner (Fig. 1B-D). Interestingly, UM-SCC1 cells were again particularly susceptible to ABT-888 alone. These results indicate that inhibition of EGFR with C225 can render cells more susceptible to the PARPi ABT-888.

### Enhanced cytotoxicity with cetuximab and ABT-888 involves activation of the intrinsic pathway of apoptosis

**Figure 2:**
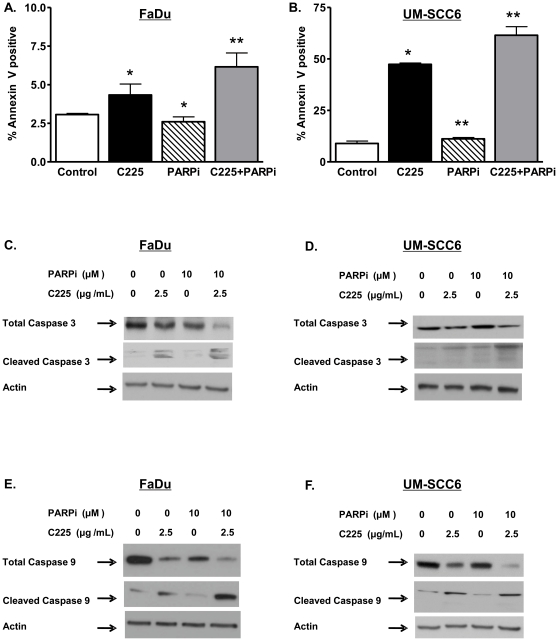
Enhanced cytotoxicity in head and neck cancer cells involves the intrinsic pathway of apoptosis. (A–B) % of apoptotic cells following combination cetuximab (C225) and ABT-888 treatment in (A) FaDu and (B) UM-SCC6 cells. Cells were treated with either vehicle or 5 µg/mL C225 for 16 hours and subsequently exposed to 10 µM ABT-888 for 24 hours. Following treatment, cells were then stained and processed for Annexin V as a marker for apoptosis. Shown is the % of Annexin V positive cells (mean +/− SEM, *p<0.01, **p<0.001 compared to vehicle control). Of note, combination of C225+PARPi was statistically different from either agent alone (*p<0.01) (C–D) C225 and ABT-888 increased apoptosis in (C) FaDu and (D) UM-SCC6 cells as evidenced by cleavage of caspase 3. (E–F) C225 and ABT-888 activated the intrinsic apoptotic pathway in (E) FaDu and (F) UM-SCC6 cells as evidenced by cleavage of caspase 9. Cells were subjected to either vehicle or 2.5 µg/mL C225 for 16 hours and subsequently subjected to ABT-888. 6 and 24 hours following the treatment period, cell lysates were harvested, and levels of total and cleaved caspase 3 (24 hours) and 9 (6 hours) were detected by Western blot analysis. A dramatic concurrent reduction in total caspase was also observed. Actin was used as a loading control. Shown is a representative Western blot of at least 3 independent experiments.

To elucidate the mechanism by which C225 and ABT-888 induce cellular cytotoxicity, we first examined activation of cellular apoptosis, since PARPi-mediated cytotoxicity has been shown to involve the apoptotic pathway [8]. We assessed cellular annexin V positivity, an early indicator of apoptosis induction. As shown in Fig. 2A and 2B, activation of apoptosis was significantly greater in both UM-SCC6 and FaDu cells with C225 and ABT-888 compared to either agent alone. Activation of apoptotic pathways ultimately leads to cleavage of caspase 3, which in turn initiates the cascade of proteolysis of integral cellular proteins and results in programmed cell death. To confirm that C225 and ABT-888 induce apoptosis in head and neck cancer cells, we assessed the levels of total and cleaved caspase 3. As shown in Fig. 2C, increased cleaved caspase 3 with a concomitant reduction of total or uncleaved caspase 3 was observed in FaDu cells following 2.5 µg/mL C225 and 10 µM ABT-888. Consistent with previous reports, C225 alone induced apoptosis in treated cells [17], [22]. A similar increase in caspase 3 cleavage was observed following C225 and ABT-888 in UM-SCC6 (Fig. 2D).

There are two major cellular apoptotic processes, consisting of the intrinsic and extrinsic pathways [23]. The extrinsic pathway is activated by proapoptotic ligand-mediated stimulation of cellular death receptors and, in turn, cleavage of caspase 8. In contrast, the intrinsic pathway is triggered by stress signals from within the cell, which ultimately results in cleavage of caspase 9.

We hypothesized that PARPi-induced apoptosis is due to intracellular stress signals from DNA damage leading to activation of the intrinsic apoptotic pathway. Consistent with this hypothesis, C225 and ABT-888 triggered cleavage of caspase 9 in FaDu (Fig. 2E) and UM-SCC6 (Fig. 2F). These data support activation of the intrinsic apoptotic pathway following C225 and ABT-888 treatment.

### Cetuximab inhibits homologous recombination and non-homologous end-joining repair

The aforementioned data supports that C225 enhances cytotoxicity with ABT-888 and activates the intrinsic pathway of apoptosis. Because lethality with PARPi has been reported to be dependent on defective DSB repair pathways [8], [10], and because EGFR has previously been shown to alter the DNA damage/response pathways, we next hypothesized that the enhanced cytotoxicity with C225 and ABT-888 was due to C225 alteration of DSB repair [13].

There are 2 major DSB repair pathways, HR- and NHEJ-mediated repair [24]. HR is a high fidelity mechanism of repair and is the preferred pathway when a homolog is present in G2 and S phase. Multiple proteins, including BRCA1, BRCA2, and Rad51, are involved in this intricate process. In contrast, NHEJ is considered an error prone system because it has to be structurally diverse to accommodate many different substrates. It occurs preferentially when a homolog is absent, outside of G2 and S phase. NHEJ is dependent on DNA-dependent protein kinase (DNA-Pk) catalytic subunit, the Ku70/80 heterodimer, and the XRCC4-ligase IV complex.

**Figure 3:**
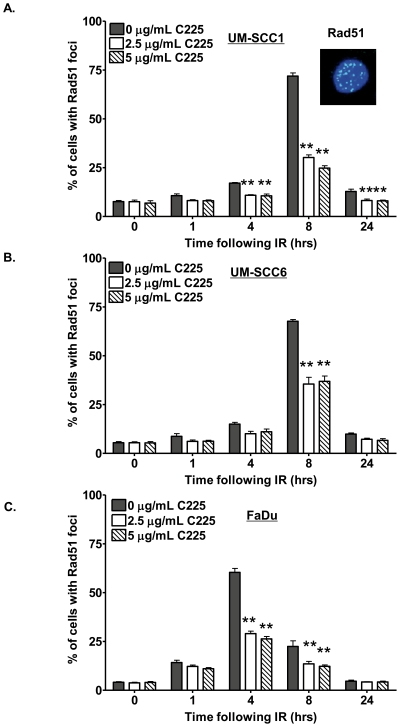
Cetuximab (C225) attenuates homologous recombination (HR) repair. C225 attenuates IR-induced Rad51 foci, well characterized markers of homologous recombination (HR)-mediated DNA DSB repair in (A) UM-SCC1, (B) UM-SCC6, and (C) FaDu cells. Cells were treated with vehicle, 2.5 µg/mL C225, or 5.0 µg/mL C225 for 16 hours and subsequently subjected to mock or 4 Gy irradiation (IR). At the indicated times following IR, cells were processed for immunofluorescence staining for Rad51 foci. Shown is the representative data of 3 independent experiments the % of cells (mean +/− SEM) with >10 foci (*p<0.05, **p<0.01 compared to vehicle at each respective time point). The inset in (A) is a representative image of UM-SCC1 cells exhibiting Rad51 foci following IR.

To test whether enhanced cytotoxicity by C225 and PARPi involves C225-mediated inhibition of DSB repair, we evaluated the effect of C225 on HR- and NHEJ-mediated DSB repair induced following γ-irradiation (IR), a potent activator of DNA DSB repair. To assess the effects of C225 on HR-mediated repair, we analyzed the kinetics of IR-induced Rad51 foci, well established markers of HR repair, at various times following 4 Gy IR. As shown in Fig. 3, IR increased the percentage of cells with Rad51 foci, peaking at 4–8 hours following IR. Consistent with our hypothesis, C225 attenuated HR by more than 50% in irradiated UM-SCC1 (Fig. 3A), UM-SCC6 (Fig. 3B), and FaDu (Fig. 3C) head and neck cancer cells. These results revealed that C225 induces a HR deficit, and the cellular susceptibility to PARPi following C225 was consistent with PARP inhibition targeting cells that are deficient in HR-mediated repair.

PARP inhibited cells have also been reported to be susceptible to inhibitors of DNA-Pk, a critical player in NHEJ [25]. This suggests that NHEJ may be an alternative DSB repair pathway besides HR to confer resistance to PARPi. Additionally, EGFR has been reported to interact and translocate with DNA-Pk to the nucleus to activate NHEJ repair processes [13], [26], [27]. It is thus possible that C225-mediated cellular susceptibility to PARPi is also due to C225 alteration of the NHEJ pathway.

**Figure 4:**
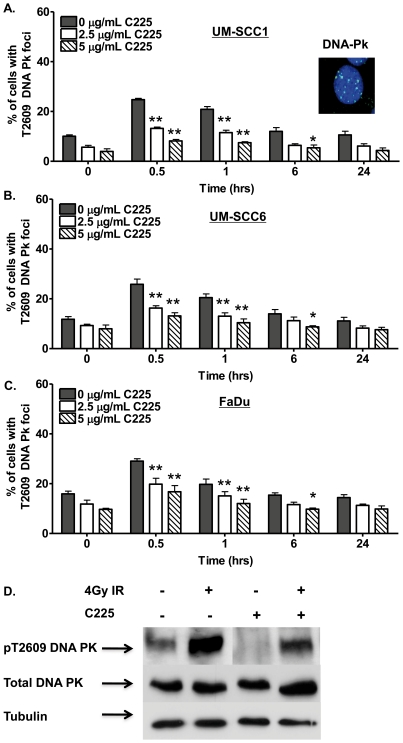
Cetuximab (C225) attenuates non-homologous end-joining (NHEJ) repair. C225 attenuates irradiation (IR)-induced DNA-Pk Thr2609 foci, well established markers of non-homologous end joining (NHEJ)-mediated DNA DSB repair in (A) UM-SCC1, (B) UM-SCC6, and (C) FaDu head and neck cancer cells. Cells were treated with vehicle, 2.5 µg/mL C225, or 5.0 µg/mL C225 for 16 hours and subsequently subjected to mock or 4 Gy IR. At the indicated time following IR, cells were processed for immunofluorescence staining for DNA-Pk Thr2609 foci. Shown is the representative data of 3 independent experiments the % of cells (mean +/− SEM) with >10 foci (*p<0.05, **p<0.01 compared to cells not exposed to C225). (D) C225 reduces phospho-Thr2609 DNA-Pk levels in UM-SCC6 head and neck cancer cells. Cells were treated with vehicle or 2.5 µg/mL C225 for 16 hours and subsequently subjected to mock or 4 Gy IR. One hour following IR, cells were processed for Western blot analysis for phospho-Thr2609 DNA Pk levels. Total DNA Pk was also analyzed and tubulin was used as loading control.

To analyze the effects of C225 on NHEJ, we assessed the kinetics of phospho-Threonine 2609 (Thr2609) DNA-Pk foci, well established markers for IR-induced NHEJ-mediated repair [28], [29], at various time points following 4 Gy IR. As expected, IR significantly increased the number of cells with phospho-Thr2609-DNA-Pk-foci at both 30 minutes and 1 hour following IR in UM-SCC1 (Fig. 4A), UM-SCC6 (Fig. 4B), and FaDu (Fig. 4C). Interestingly, the addition of C225 significantly attenuated this response by more than 30% in all cell lines examined.

EGFR has also been shown to phosphorylate and activate DNA-Pk [13], [26], [27]. To determine whether inhibition of NHEJ by C225 is due to reduced phosphorylation of DNA-Pk, we next examined levels of phospho-DNA-Pk following C225. As shown in Fig. 4D, C225 reduced DNA-Pk phosphorylation without altering total DNA-Pk in UM-SCC1, UM-SCC6, and FaDu cells, which is consistent with C225-mediated inhibition of NHEJ-mediated repair.

Taken together, these data indicate that C225 induces a DSB repair deficiency of the 2 major DSB repair pathways, NHEJ and HR, and enhanced cytotoxicity by C225 with PARPi is due to inhibition of both major DSB repair pathways.

### EGFR inhibition increases DNA damage

**Figure 5:**
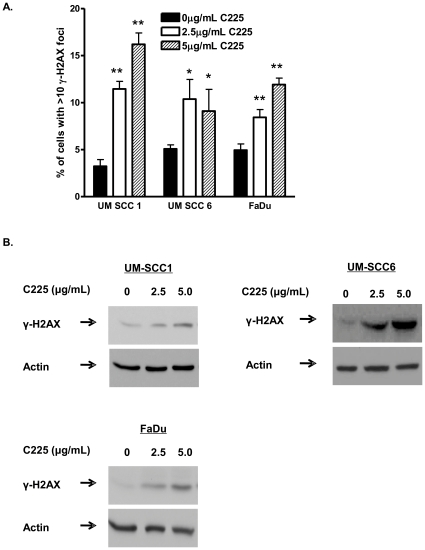
Cetuximab (C225) increases DNA damage by inhibiting DNA double strand break (DSB) repair in head and neck cancer cells. (A) C225 increases the number of cells with DSBs as evidenced by γ-H2AX foci, a commonly used marker for DSBs. Shown is the representative data of 3 independent experiments the % of cells (mean +/− SEM) with >10 foci (*p<0.05, **p<0.01 compared to vehicle control). (B) C225 increases γ-H2AX protein levels in treated cells. UM-SCC1, UM-SCC6, and FaDu cells were treated with vehicle, 2.5 µg/mL C225, or 5.0 µg/mL C225 for 16 hours. Following the treatment period, cells were processed for (A) immunofluorescence staining for γ-H2AX foci or (B) western blot analysis for γ-H2AX levels. Shown is the representative Western blot of 3 independent experiments.

C225 induces a DSB repair deficiency in head and neck cancer cells (Fig. 3 and 4). We hypothesized that C225-treated cells should exhibit increased markers of DNA DSBs. To assess DNA DSBs, we examined the effect of C225 on γ-H2AX foci, which are well documented markers of DNA DSBs [30], in UM-SCC1, UM-SCC6, and FaDu cell lines. As shown in Fig. 5A, all cell lines exhibited significantly increased DNA damage following C225 as demonstrated by increased percentage of cells with γ-H2AX foci in a dose-dependent manner. This was confirmed via Western blot analysis, which revealed increased γ-H2AX levels following various doses of C225 in UM-SCC1, UM-SCC6, and FaDu cells (Fig. 3B). These results indicated that inhibition of EGFR with C225 increases DNA DSB damage in treated cells, which is consistent with C225-induced inhibition of DSB repair.

### Combination cetuximab and ABT-888 generates persistent DNA damage

**Figure 6:**
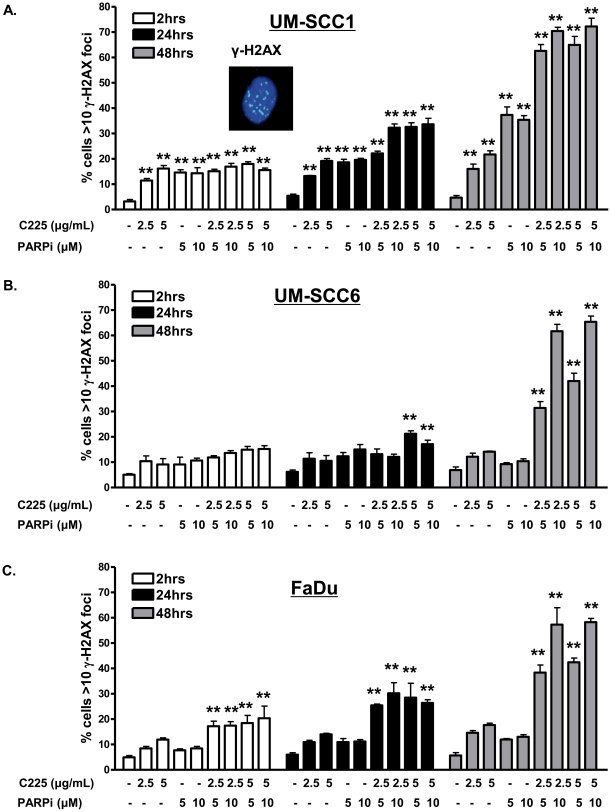
Combination cetuximab (C225) and ABT-888 induces persistent DNA double strand break damage in head and neck cancer cells. (A–C) DNA damage 2, 24, and 48 hours following vehicle, C225, PARPi, or both C225+PARPi was assessed by γ-H2AX foci in (A) UM-SCC1, (B) UM-SCC6, and (C) FaDu cells. Cells were treated with vehicle or various doses of C225 for 16 hours and subsequently exposed to vehicle or various doses of ABT-888. At the indicated times following PARP inhibition, cells were processed for immunofluorescence staining for γ-H2AX foci. Shown is the representative data of 3 independent experiments the % of cells (mean +/− SEM) with >10 foci (*p<0.05, **p<0.01 compared to vehicle control at each respective time point).

PARPi inhibits the base excision repair pathway responsible for the resolution of DNA single strand breaks (SSBs). SSBs which persist in dividing cells are ultimately converted to DSBs and repaired by HR-mediated repair. Given that C225 reduces DSB repair capacity and that C225 enhances cytotoxicity with ABT-888, we hypothesized that the combination C225 and ABT-888 would result in further persistent DNA DSB damage. To evaluate this, we performed a time course analysis of γ-H2AX foci with vehicle, C225 alone, ABT-888 alone, or combination C225 and ABT-888. As shown in Fig. 6, compared to vehicle control, C225 alone as expected induced 2–3 fold the % of cells with increased DNA damage in UM-SCC1 (Fig. 6A), UM-SCC6 (Fig. 6B), and FaDu (Fig. 6C) head and neck cancer cells. Interestingly, the combination of C225 and ABT-888 resulted in a significantly greater number of cells with persistent DNA damage in all cell lines examined (Fig. 6). Moreover, the UM-SCC1 cells (Fig. 6A), which exhibited exquisite sensitivity to ABT-888 alone, also had persistent DNA damage with ABT-888 alone. In contrast, in UM-SCC6 (Fig. 6B) and FaDu (Fig. 6C) cells, ABT-888 alone did not result in significant increase in cells with evident DNA DSB damage. These results demonstrate that cytotoxicity from C225 and PARPi may be due to the inability of treated cells to resolve DNA DSBs, the most critical lesion in cells.

### Effects of cetuximab and ABT-888 on DNA damage and repair is not due to cell cycle redistribution

**Figure 7:**
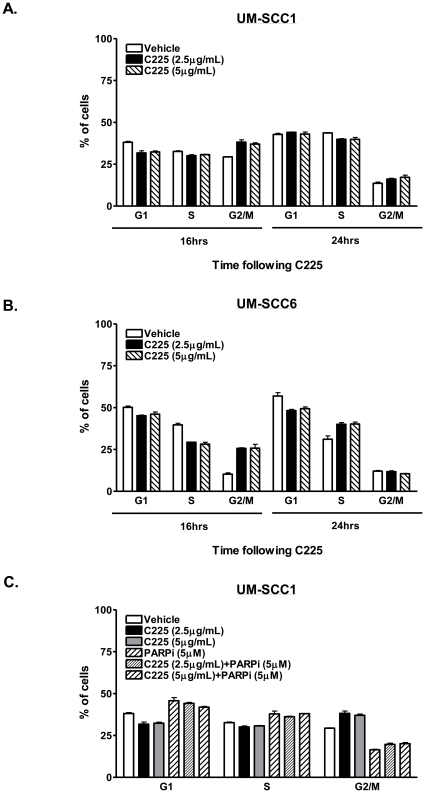
Effects of cetuximab (C225) and ABT-888 are not due to cell cycle redistribution. Cell cycle distribution of (A) UM-SCC1 or (B) UM-SCC6 cells following treatment with vehicle or C225. (C) Cell cycle distribution of UM-SCC1 cells following vehicle, C225, ABT-888, or combination C225 and ABT-888. Cells were treated with vehicle or various doses of C225 for 16 hours and subsequently exposed to vehicle or 5 µM of ABT-888. Forty-eight hours following PARP inhibition, cells were processed for cell cycle analysis by flow cytometry. Shown is the representative cell cycle distribution (mean +/− SEM) of at least 2 independent experiments performed in triplicate.

DNA repair pathways, in particular HR, can be dependent on the cell cycle. Additionally, EGFR is involved in cell proliferation pathways, and inhibition of EGFR has been shown to induce cell cycle redistribution [4], [31], [32]. It is possible that inhibition of HR by C225 may be an indirect effect of increased cellular accumulation in the G1 phase of the cell cycle. We thus investigated the cell cycle distribution of cells treated with vehicle or C225 to rule out cell cycle effects as a potential confounder by which C225 alters DNA DSB repair. As shown in Fig. 7, there is an absence of any cell cycle redistribution following treatment in UM-SCC1 (Fig. 7A) or UM-SCC6 (Fig. 7B) to account for C225-mediated reduction in DSB repair at the time points at which HR repair was measured.

ABT-888 has also been reported to cause senescence when combined with radiation in breast cancer cells [33]. Additionally, other PARPi can induce G2/M accumulation of cells [34]. Thus, to assess cell cycle changes as another potential mechanism of enhanced cytotoxicity, cell cycle distribution following combination C225 and ABT-888 was performed in UM-SCC1 cells. As shown in Fig. 7C, no cell cycle redistribution was observed. These results demonstrated that C225-induced attenuation of DSB repair pathways and the subsequent enhanced cytotoxicity with ABT-888 were not due to cell cycle effects.

## Discussion

In this study, we demonstrate that C225, an inhibitor of EGFR, augments cellular susceptibility to the PARPi ABT-888 in head and neck cancer cells. The mechanism of enhanced cytotoxicity involved C225-mediated attenuation of the two major DNA DSB repair pathways, NHEJ and HR, which leads to the persistence of DNA damage following PARPi and the subsequent activation of the intrinsic pathway of apoptosis. Thus, the combination of C225 and the PARPi ABT-888 can be an innovative treatment strategy to potentially improve outcomes in head and neck cancer patients. This combination of C225 and ABT-888 may be particularly exciting for regimens that include other DNA damaging agents such as radiation.

The EGFR has been implicated in a number of cellular processes, including cell proliferation and survival, angiogenesis, and DNA damage response and repair. Specifically, with regards to DNA damage response, EGFR has been shown to translocate to the nucleus and interact with DNA-Pk to activate NHEJ [13], [26], [27]. Activated EGFR can also increase Rad51 foci and expression levels to regulate HR [35]. These actions by EGFR have been attributed to resistance of EGFR amplified/mutated tumors to DNA damaging agents and provide rationale for targeted inhibition of EGFR.

In support of a role of EGFR in the DNA damage and repair pathways, C225, which inhibits EGFR, attenuates the two major DNA DSB repair pathways, HR and NHEJ, by altering Rad51 and DNA-Pk foci levels, respectively. C225 also inhibited DNA-Pk phosphorylation. As PARPi has been shown to target HR-deficient cells, the actions of C225 on HR-mediated repair provide rationale for why the novel combination of C225 and PARPi enhances cytotoxicity in head and neck cancer cells [8], [9]. Additionally, PARP inhibited cells have been shown to be sensitized to inhibitors of the NHEJ pathway, suggesting that NHEJ may also be a backup pathway of unresolved SSBs [25]. This may also explain the dramatic cytotoxicity observed in C225 and PARPi treated cells. Furthermore, as C225 induces both a NHEJ and HR repair deficiency, the combination of C225 with PARPi leads to a high proportion of treated cells with persistent DSBs. Given these observations, cells exposed to C225 and PARPi should be exquisitely susceptible to other DNA damaging agents, such as radiation. This is an area of active investigation in our laboratory.

C225 and PARPi also enhanced apoptosis, which is consistent with previous reports of PARPi-mediated cytotoxicity [8]. We found that this apoptosis was a result of activation of the intrinsic pathway. It is worth noting that the magnitude of regulation of apoptosis does not reach the levels of cytotoxicity measured by colony formation assays. Multiple pathways other than apoptosis could affect the colony-forming abilities of cells, such as inhibition of cell proliferation, cell cycle arrest, mitotic catastrophe, and autophagy. This discrepancy may also be explained by the notion that contrary to analysis of foci or immunoblotting, which demonstrates the effect at a snap shot in time, the colony formation assay reflects multiple mechanisms of cell death over a period of 3 weeks. As multiple signaling pathways are involved in regulation and determination of the fate of cell death or survival, our data suggests that inhibition of EGFR may be one part of the complicated cell signaling/DNA damage repair network, and may contribute only partly to the overall effect of cell susceptibility to DNA damage. It is, thus, likely that PARPi and EGFR inhibition might regulate multiple cytotoxic pathways. For example, ABT-888 in combination with radiation has also been shown to induce autophagic cell death in lung cancer cells [36]. Thus, other mechanisms of cell death, including autophagy, cannot be ruled out.

Since PARP is a SSB DNA repair enzyme, treatment with the PARPi ABT-888 is expected to inhibit SSB repair and thus increase basal levels of SSBs. Addition of C225 results in further DNA damage. The increased DNA damage observed at longer time points may be due to persistent DSBs or the result of additional DNA-cuts as a consequence of conversion of SSBs to DSBs during attempted DNA repair or collapsed replication forks. This is supported by the increased % of cells with γ-H2AX foci at later time points. Alternatively, activation of cell death processes such as apoptosis could also induce markers of DNA damage.

Interestingly, the UM-SCC1 head and neck cancer cells exhibit susceptibility to PARPi alone. These cells are not inherently DSB repair deficient, as assessed by IR-induced Rad51 and DNA-Pk foci. However, PARPi alone induces persistent γ-H2AX foci, suggestive of the presence of persistent DSBs. It is intriguing to postulate that other molecular determinants of PARPi susceptibility independent of inherent DNA repair defects must exist. One of several possibilities is the recently reported increased occupancy by repressive E2F4/p130 complexes of the BRCA1 and RAD51 promoters in the presence of PARPi, thus increasing cellular susceptibility to oxidative damage by suppressing the backup DSB repair pathways [37].

In the last several years, the association between human papilloma virus (HPV) and head and neck cancer has been solidified [38], [39]. Interestingly, HPV associated head and neck cancers exhibit a better prognosis and appear to respond better to chemoradiation [40]. It is postulated that this is due to HPV oncoproteins and alteration of the DNA damage/response pathways [41], [42]. Interestingly, E7 expression has been shown to disrupt E2F4 and p130 repressive activity and prevented PARPi-mediated downregulation of BRCA1 and Rad51 [37]. However, interaction between all the HPV-oncogenes and the DNA damage response may result in different susceptibilities to DNA damage. Thus, it would be interesting to assess the susceptibility of HPV-associated tumors to PARPi.

Our study demonstrates that inhibition of EGFR with C225 enhances cytotoxicity with the PARPi ABT-888 in head and neck cancer cells via C225-mediated disruption of the HR- and NHEJ-mediated DSB repair pathways. These results warrant future studies to compare efficacy versus traditional chemotherapy. More importantly, as maintaining quality of life has become an area of emphasis in oncology, the use of targeted agents such as C225 and ABT-888 may further improve the therapeutic ratio. Lastly, this strategy may also be feasible in other tumors with aberrant EGFR signaling, such as brain and lung cancers.

## Materials and Methods

### Cell culture

The human head and neck squamous carcinoma cell lines UM-SCC1 and UM-SCC6 were obtained courtesy of Dr. Thomas E Carey (University of Michigan, Ann Arbor, MI). They were maintained in DMEM (GIBCO, Invitrogen) supplemented with 10% fetal bovine serum (FBS, Atlanta Biologicals) and 1% Penicillin/Streptomycin (Invitrogen). The human head and neck squamous carcinoma cell line FaDu (HTB-43) was obtained from ATCC (Manassas, VA) and was maintained in RPMI-1640 (GIBCO, Invitrogen) supplemented with 10% FBS. The PARP inhibitor ABT-888 (Enzo Life Sciences) and cetuximab (C225, Bristol Myers Squibb) were utilized in our study.

### Cell Viability

Cell viability was measured using the ATP-lite 1 step luminescence assay (Perkin Elmer) following the manufacturer's directions. Briefly, 1000 cells in exponential phase were seeded per well in a 96 well plate and treated with cetuximab (C225, Bristol Myers Squibb) or vehicle for 16 hours, after which the PARP inhibitor ABT-888 (10 µM, Enzo Life Sciences) was added. Cells were pretreated with C225 to mimic the loading dose of C225 that is given as one standard regimen for head and neck cancer therapy. Relative ATP levels were measured 24 hours later using Perkin Elmer luminometer.

### Clonogenic survival assay

Cell survival was evaluated by the colony formation assay in the head and neck squamous cell carcinoma cell lines following 2.5 µg/mL C225 and various doses of ABT-888 (1 µM-20 µM) as previously described [43]. Briefly, cells in exponential phase were seeded and treated with either C225 or vehicle. Sixteen hours following C225 treatment, the indicated doses of ABT 888 (or vehicle) was added. 24 hours post the first dose of ABT-888, cells were subjected to a second dose and plates were left undisturbed. Three weeks following initial treatment, colonies were fixed with 70% ethanol, stained 1% methylene blue and number of positive colonies were counted (>50 cells). Survival fraction was calculated as follows: (number of colonies for treated cells/number of cells plated)/(number of colonies for corresponding control/number of cells plated). Experiments were performed in triplicate.

### Analysis of apoptosis

8×10^4^ cells were seeded in each well of a 6-well plate and treated with C225 or vehicle control. Sixteen hours post C225 treatment, 10 µM ABT-888 or vehicle was added. Forty hours post-C225 treatment both attached and floating cells were collected in 12×75 mm culture tubes. Annexin V-FITC Apoptosis Detection kit (BioVison Research Products; catalog # K101-400) was used according to manufacturer's instructions to measure percentage of apoptotic cells by FACScan using CellQuest. Control samples included 1× Binding Buffer only, Annexin V-FITC only, and propidium iodide (PI) only. Experiments were performed in triplicate.

### Immunofluorescence

To evaluate DSB repair capacity, head and neck cell lines were cultured and seeded on sterile cover slips, exposed to various doses of C225 for sixteen hours. To assay DNA Pk and Rad51 activity, cells were subsequently treated with mock or 4 Gy γ-IR using an X-ray irradiator (Kimtron Inc., Woodbury, CT). Following the treatment period, cells were fixed at the indicated time points. The same procedure was followed to assay the effect of C225 on DNA damage as measured by the formation of γ-H2AX foci, except that no radiation treatment was utilized. To measure the effect of C225 and PARPi combination on DNA damage, sixteen hours following C225 treatment, cells were exposed to various doses of ABT-888 and fixed at the indicated time points and immunohistochemistry was performed as previously described [44] with slight modification. Briefly, cells were rinsed in phosphate buffered saline (PBS) and incubated for 5 minutes at 4°C in ice-cold cytoskeleton buffer (10 mM Hepes/KOH, pH 7.4, 300 mM sucrose, 100 mM NaCl, 3 mM MgCl_2_) supplemented with 1 mM PMSF, 0.5 mM sodium vandate and proteasome inhibitor (Sigma, 1∶100 dilution) followed by fixation in 70% ethanol for 15 minutes. The cells were blocked and incubated with primary antibodies [1∶500 dilution, DNA Pkcs phospho T2609, Genetex, catalog # GTX-24194; Rad 51 (H-92), Santa Cruz Biotechnology, catalog # sc-8349, phospho H2AX Ser139, Millipore, catalog # MI-07-164]. Secondary antibodies include anti-mouse Alexa Fluor 488–conjugated antibody (1∶2000; Invitrogen) or anti-rabbit Alexa Fluor 594–conjugated antibody (1∶2000 dilution; Invitrogen). DAPI (Invitrogen, catalog # D21490) was used for nuclear staining. The cover slips were subsequently mounted onto slides with mounting media (Aqua poly mount, Polysciences, Inc. catalog # 18606) and analyzed via fluorescence microscopy (Carl Zeiss, Thornwood, NY). Positive and negative controls were included on all experiments. A total of 500 cells were assessed. For foci quantification, cells with greater than 10 foci were counted as positive according to the standard procedure.

### Immunoblotting

Cell lysates were prepared using radioimmunoprecipitation lysis buffer (150 mM NaCl, 50 mM Tris, pH 8.0, 5 mM EDTA, 0.5% sodium deoxycholate, 0.1% SDS, 1.0% Nonidet P-40) with protease and phosphatase inhibitor cocktails (Sigma) and subjected to SDS-PAGE analysis. The following antibodies were used at dilutions recommended by the manufacturer: cleaved caspase 3 (Asp 175) (Cell Signalling Technology, catalog # 9664), total caspase 3 (Cell Signalling Technology, catalog # 9662), cleaved caspase 9 (Asp 330) (Cell Signalling Technology, catalog # 9501), total caspase 9 (Cell Signalling Technology, catalog # 9502), phospho H2AX Ser139 (Millipore, catalog # MI-07-164), DNA Pkcs (Santa Cruz Biotechnology, catalog # sc-1552), DNA Pkcs phospho T2609 (Genetex, catalog # GTX-24194). β-Actin (Santa Cruz Biotechnology, catalog # sc47778) or tubulin (Santa Cruz Biotechnology, catalog # sc-53646) levels were also analyzed as loading control.

### Cell cycle analysis

Cell cycle distribution was measured as previously described [45]. 2×10^5^ cells were seeded in 100 mm^2^ dishes and treated with 2.5 µg/mL C225 or vehicle. 16 hours post-C225 treatment, 10 µM ABT 888 or vehicle was added. Cells were collected and fixed at different time points, treated with RNAse (Sigma, catalog # R-4875), stained with propidium iodide (PI), and read on FACSCalibur using CellQuest. Data was analyzed using ModFit LT by Verity Software Inc.

### Statistical analysis

The data were analyzed via analysis of variance (ANOVA) followed by a Bonferroni post test using GraphPad Prism version 4.02 (GraphPad Software, San Diego, CA). Data presented as average +/− standard error of mean.
